# In Vivo Bioluminescence Imaging for Longitudinal Monitoring of Inflammation in Animal Models of Uveitis

**DOI:** 10.1167/iovs.16-20824

**Published:** 2017-03

**Authors:** Michal B. Gutowski, Leslie Wilson, Russell N. Van Gelder, Kathryn L. Pepple

**Affiliations:** 1 Department of Ophthalmology, University of Washington, Seattle, Washington, United States; 2 Department of Biological Structure, University of Washington, Seattle, Washington, United States; 3 Department of Pathology, University of Washington, Seattle, Washington, United States

**Keywords:** animal model, uveitis, bioluminescence imaging, inflammation, IVIS

## Abstract

**Purpose:**

We develop a quantitative bioluminescence assay for in vivo longitudinal monitoring of inflammation in animal models of uveitis.

**Methods:**

Three models of experimental uveitis were induced in C57BL/6 albino mice: primed mycobacterial uveitis (PMU), endotoxin-induced uveitis (EIU), and experimental autoimmune uveitis (EAU). Intraperitoneal injection of luminol sodium salt, which emits light when oxidized, provided the bioluminescence substrate. Bioluminescence images were captured by a PerkinElmer In Vivo Imaging System (IVIS) Spectrum and total bioluminescence was analyzed using Living Image software. Bioluminescence on day zero was compared to bioluminescence on the day of peak inflammation for each model. Longitudinal bioluminescence imaging was performed in EIU and EAU.

**Results:**

In the presence of luminol, intraocular inflammation generates detectable bioluminescence in three mouse models of uveitis. Peak bioluminescence in inflamed PMU eyes (1.46 × 10^5^ photons/second [p/s]) was significantly increased over baseline (1.47 × 10^4^ p/s, *P* = 0.01). Peak bioluminescence in inflamed EIU eyes (3.18 × 10^4^ p/s) also was significantly increased over baseline (1.09 × 10^4^ p/s, *P* = 0.04), and returned to near baseline levels by 48 hours. In EAU, there was a nonsignificant increase in bioluminescence at peak inflammation.

**Conclusions:**

In vivo bioluminescence may be used as a noninvasive, quantitative measure of intraocular inflammation in animal models of uveitis. Primed mycobacterial uveitis and EIU are both acute models with robust anterior inflammation and demonstrated significant changes in bioluminescence corresponding with peak inflammation. Experimental autoimmune uveitis is a more indolent posterior uveitis and generated a more modest bioluminescent signal. In vivo imaging system bioluminescence is a nonlethal, quantifiable assay that can be used for monitoring inflammation in animal models of uveitis.

Animal models have had a key role in studying mechanisms of ocular inflammation, and are important for preclinical testing of new therapies.^1^ Despite the importance of these models, in vivo clinical scoring of inflammation in animal models can be challenging. For example, the variability in disease severity generated in models of experimental autoimmune uveitis (EAU) by different antigens and strains has led to the creation of at least four different in vivo scoring systems.^2–5^ The small size of the mouse eye also contributes to the technical challenges of reliable clinical scoring. To achieve a higher resolution analysis of endpoints, postmortem histologic scoring systems or flow cytometry analysis of infiltrating inflammatory cells can be performed. However, these assays are not compatible with longitudinal evaluation in a single animal, and necessitate the use of cohorts of animals at multiple time points. The optimal scoring system would use a quantifiable assay that could be repeated in individual animals to accurately track the course of inflammation and measure the impact of an intervention on the spontaneous course of inflammation.

In vivo bioluminescence imaging has become a widely used tool for studying biological processes in small laboratory animals.^6,7^ Bioluminescence differs from fluorescence in the way that light is generated. Fluorescence is generated when a fluorophore absorbs light of a shorter wavelength (higher energy) and emits light of a longer wavelength (lower energy). In contrast, bioluminescence does not require incident light. Instead, photons are generated secondary to a chemical reaction occurring within a living organism. One method for generation of bioluminescence uses the ability of luminol (5-amino-2,3-dihydro-1,4-phthalazine-dione) to emit light (λ_max_ = 425 nm) when exposed to an oxidizing agent like hypochlorous acid (a product of myeloperoxidase activity within activated neutrophils).^8,9^ The light produced by this reaction can be captured and quantified using commercially available charged couple device (CCD) camera systems. Systemic administration of luminol has been used to measure in vivo inflammation in models of dermatitis,^8^ arthritis,^10^ and spinal cord injury.^11^ Therefore, we proposed the following series of pilot experiments to determine the feasibility of using luminol-based bioluminescence imaging to detect and quantify ocular inflammation. To determine if the technique would have widespread use, we tested the method in three different models of uveitis, EAU,^3^ endotoxin-induced uveitis (EIU),^12,13^ and primed mycobacterial uveitis (PMU).^14,15^

## Methods

### Animals and Uveitis Induction

Female C57BL/6 albino mice (*n* = 13) were purchased from Jackson Laboratories (Bar Harbor, ME, USA) and maintained with standard chow and water ad libitum under specific pathogen-free conditions. The animal study protocol was approved by the Animal Care and Use Committee of the University of Washington (animal study protocol #4184-04) and was compliant with the ARVO Statement for the Use of Animals in Ophthalmic and Vision Research. Primed mycobacterial uveitis was generated as described previously^14,15^ with modifications of the protocol for use in mice. Briefly, animals received subcutaneous injection of 100 μg killed *Mycobacterium tuberculosis* H37Ra antigen (#231141; Difco Laboritories, Detroit, MI, USA) in 0.1 cc of an emulsion of incomplete Freund's adjuvant (#263910; Difco Laboritories). Seven days later (designated as day zero) the right eye of each animal received an intravitreal injection of 5 μg (*n* = 4) or 3.5 μg (*n* = 1) of a suspension of killed *Mycobacterium tuberculosis* H37Ra antigen in 1 μl of PBS. Endotoxin-induced uveitis (EIU) was induced as described previously by intravitreal injection of 1 μL of a 125 ng/μL lipopolysaccharide (LPS) (Invivogen, San Diego, CA, USA) in PBS into the right eye.^13^ Experimental autoimmune uveitis was generated as described previously with subcutaneous injection of 500 μg interphotoreceptor retinoid binding protein peptide 1–20 (IRBP^1–20^; GPTHLFQPSLVLDMAKVLLD; Peptide 2.0, Chantilly, VA, USA) in 0.1 cc complete Freund's adjuvant (2.5 mg/mL H37Ra in incomplete Freund's Adjuvant) on day 0.^3^ Experimental autoimmune uveitis animals also received 0.15 μg intraperitoneal pertussis toxin (Sigma-Aldrich Corp., St. Louis, MO, USA) on days 0 and 2.

### Bioluminescence Imaging System, Image Acquisition, and Image Analysis

Bioluminescence images were captured using the In Vivo Imaging System (IVIS) Spectrum (Perkin Elmer, Santa Clara, CA, USA) and analyzed using IVIS imaging software (Perkin Elmer). Ten minutes before imaging animals received an intraperitoneal (IP) injection of 200 mg/kg luminol sodium salt (Sigma Life Science, St. Louis, MO, USA). Anesthesia was provided with inhaled isoflurane, eyes were dilated with phenylephrine (2.5%; Akorn, Inc., Lake Forest, IL, USA), and corneal protection was provided using Genteal (Alcon Laboratories, Inc., Fort Worth, TX, USA). Imaging was performed on all animals on day zero before uveitis induction. For PMU, imaging was repeated on day 2. For EIU imaging was repeated at 18 (*n* = 4 mice) and 48 (*n* = 1 mouse) hours. For EAU, imaging was repeated on days 15 and 21. Animals were positioned on the IVIS warming stage in the left lateral decubitus position with the ocular surface directly facing the camera sensor. Positioning was maintained using a Costar 50 ml reagent reservoir (Corning, Corning, NY, USA) with one end removed to allow nose cone positioning for continuous inhaled isoflurane anesthesia. In vivo imaging system imaging parameters were determined using standard optimization protocols.^16^ Briefly, animals were imaged using field of view “A,” subject height 1.5 cm, with medium binning for five minutes. Two images were acquired for right eyes (from 10–15 and 15–20 minutes after luminol injection). Mice then were positioned to the right lateral decubitus position and left eye images were acquired from 21 to 26 minutes after luminol injection. Right eye total bioluminescence is determined as the sum of the background-subtracted ocular region of interest (ROI) flux from two consecutive 5-minute imaging windows (5–10 and 10–15 minutes). Left eye total bioluminescence is determined as two times the background-subtracted ocular ROI flux from the 21- to 26-minute imaging window. Average bioluminescence at baseline and at peak inflammation was compared by paired *t*-tests using Prism 6 GraphPad software (San Diego, CA, USA). Before *t*-test analysis, normality of the differences of paired data was tested by Shapiro-Wilk analysis with failure to reject the null hypothesis.

### Optical Coherence Tomography (OCT) System, Image Acquisition, and Analysis

Optical coherence tomography images were acquired using the Bioptigen Envisu R2300 (Bioptigen, Inc., Morrisville, NC, USA). Anesthesia was provided with 6.9 mg/kg ketamine/xylazine IP (1% solution; Ketamine, Ketaset 100 mg/mL; Zoeitis, Inc. Kalamazoo, MI, USA; Xylazine, AnaSed 20 mg/mL; Lloyd Laboratories, Shenandoh, IA, USA). Eyes were dilated with phenylephrine (2.5%, Akorn, Inc.) and corneal protection provided by Genteal (Alcon Laboratories, Inc.). Animals were wrapped in warming gauze and placed in the prone position in the Bioptigen mouse imaging cassette. For the anterior chamber, 3.6 × 3.6 mm images (1000 A-lines/B-Scan × 400 B-scans) were captured using a Bioptigen 12 mm telecentric lens (product # 90-BORE-G3-12; Bioptigen, Inc.). For retinal imaging, 1.6 × 1.6 mm images (1000 A-lines/B-scan × 200 B-scans) were captured using the Bioptigen mouse retina lens (product # 90-BORE-G3-M; Bioptigen, Inc.). A manual grader scored OCT images. For anterior chamber (AC) images, three B-scan images per animal per time point were analyzed. The number of free-floating AC cells were counted on each image and then averaged to provide an AC cell count/B-scan for each animal. The presence or absence of a hypopyon was noted and given a value of 1 to 4+ based on size with 4+ indicating depth of 1/2 the anterior chamber. For retina/vitreous images, three B-scan images centered on the optic nerve per animal per time point were analyzed. The number of vitreous cells was counted on each image and then averaged to provide an average vitreous cell count/B-scan for each animal. If individual cells could not be counted in the vitreous, the presence of a vitreous consolidation was noted and given a value of 1 to 4+ based on area of the consolidation, with 1+ indicating a size less than or equal to the cross-sectional area of the optic nerve head, 2+ indicating a size >1+ but less than half the area of the vitreous, 3+ indicating a size greater than half the area of the vitreous, but not completely filling the vitreous, and 4+ indicating a size completely filling the vitreous. Vitreous images were not obtained for PMU animals.

### Histology

Postmortem eyes were collected and placed in 10% neutral buffered formalin (Sigma Life Science), and embedded in paraffin blocks by standard protocols. Sections (4 μm) were stained with hematoxylin and eosin (H&E). Three sections were scored and averaged. For EAU eyes, inflammation was assigned a score of 0 to 4 as previously described.^3^ For EIU and PMU the number of cells in the AC and vitreous were counted on three sections and averaged.^12,17^

## Results

**Figure 1:**
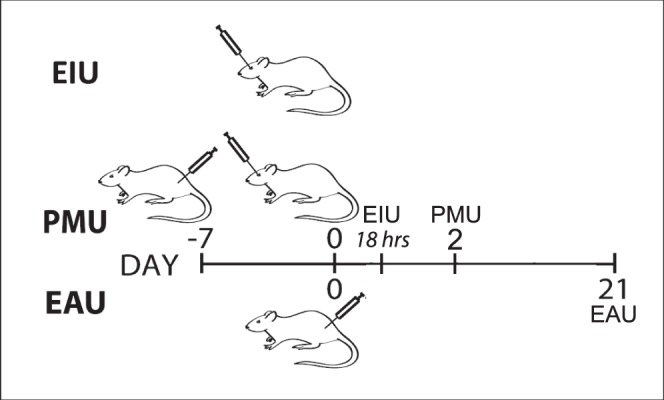
Time course of inflammation for three uveitis models. Endotoxin-induced uveitis peaks 18 hours after intravitreal LPS injection and resolves by 48 hours. Primed mycobacterial uveitis is initiated with a subcutaneous injection of killed mycobacterial injection 7 days before intravitreal injection of killed mycobacterial extract and peaks 2 days later. Experimental autoimmune uveitis peaks at day 21 after subcutaneous injection of IRBP peptide in complete Freund's adjuvant.

**Figure 2:**
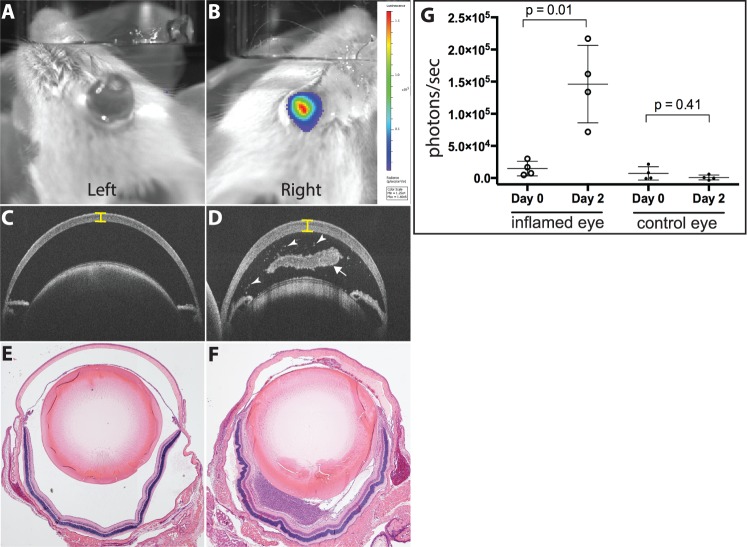
Luminol bioluminescence detection of inflammation in PMU. Day 2 bioluminescence images of (**A**) an uninflamed left eye and (**B**) an inflamed right eye. Color scale reflects photon density (*red* for highest density). Total bioluminescence = 1.5 × 10^5^ p/s in the inflamed eye region of interest. (**C**) Left and (**D**) right eye OCT on day 2. The inflamed eye (**D**) demonstrates corneal edema (*bracket*), AC cell (*arrowheads*), and pupillary membrane (*arrow*). Histology of the (**E**) left and (**F**) right eyes on day two verifies the absence (**E**) and presence (**F**) of inflammation. (**G**) Total bioluminescence on day 2 from right and left eyes. The difference between the average bioluminescence of inflamed eyes on day 2 (1.46 × 10^5^ p/s) and at baseline (1.47 × 10^4^ p/s) was significant (*P* = 0.01).

**Figure 3:**
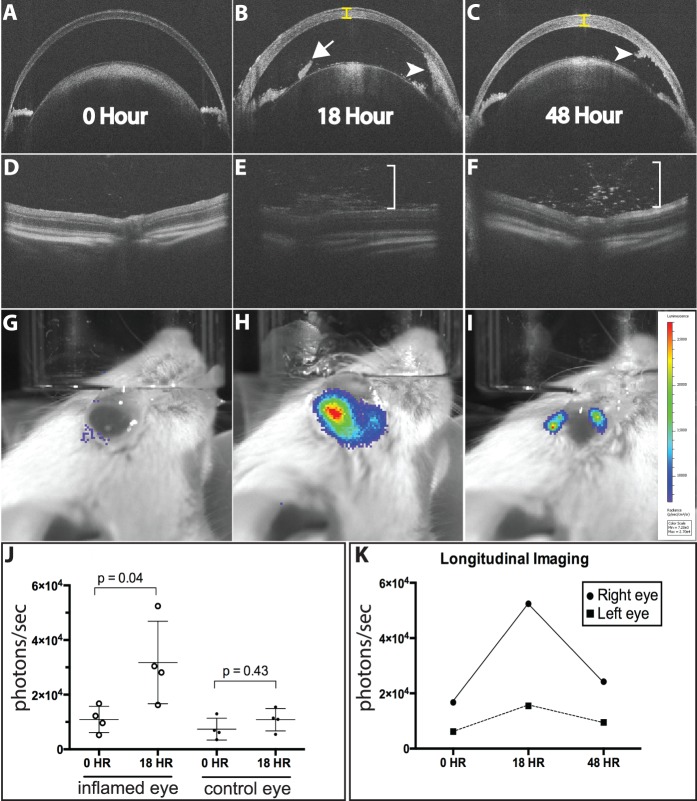
Luminol bioluminescence detection of inflammation in EIU. Optical coherence tomography of the anterior chamber (**A**–**C**) and the vitreous and retina (**D**–**F**) at times 0, 18, and 48 hours. Corneal edema (*yellow brackets*), small pupillary membrane (*arrow*), and hypopyon (*arrowhead*), and vitritis (*white bracket*) are seen at 18 hours. At 48 hours, the hypopyon is decreased in size (*arrowhead*), and there is less vitritis. (**G**–**I**) Bioluminescence images at (**G**) time 0, (**H**) 18 hours, and (**I**) 48 hours. (**J**) Bioluminescence at 18 hours from right and left eyes. The difference between the average total bioluminescence of all right eyes at time zero (1.09 × 10^4^ p/s) and at 18 hours (3.18 × 10^4^ p/s) was significant (*P* = 0.04). Longitudinal imaging performed demonstrated peak bioluminescence at 18 hours, and decline in bioluminescence by 48 hours.

**Figure 4:**
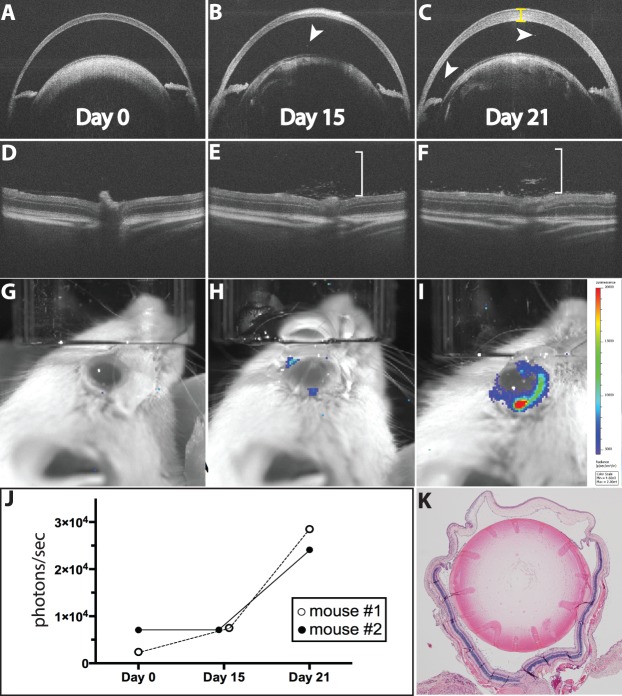
Luminol bioluminescence detection of inflammation in EAU. Optical coherence tomography of the anterior chamber (**A**–**C**) and the vitreous and retina (**D**–**F**) at days 0, 15, and 21. Corneal edema (*yellow brackets*), rare AC cells (*arrowheads*), and vitritis (*white bracket*) develop over the course of inflammation. Bioluminescence images on days (**G**) 0, (**H**) 15, and (**I**) 21. Ocular bioluminescence at day 21 = 2.9 × 10^4^ p/s. (**J**) Change in bioluminescence in 2 animals. (**K**) Postmortem histology after imaging on 21 confirms inflammation for the eye shown in (**A**–**I**).

To test the ability of luminol to detect intraocular inflammation, three models of uveitis were generated in C57BL/6 albino mice (see Fig. 1). Four mice were used in PMU experiments (Fig. 2), in EIU experiments (Fig. 3), and in EAU experiments. However, only the two EAU animals that demonstrated inflammation by OCT and histology are shown in Figure 4. Primed mycobacterial uveitis is a model of unilateral anterior and intermediate uveitis that was initially described in rabbits and used for preclinical testing of the fluocinolone implant Retisert.^15,18^ This model subsequently has been described in Lewis rats.^14^ In the mouse model of PMU, intraocular inflammation is generated by subcutaneous injection of a killed mycobacterial extract in incomplete Freund's adjuvant 7 days before unilateral intravitreal injection of the same mycobacterial extract in PBS. This generates a robust anterior chamber reaction in one eye that can be identified with anterior segment OCT (Fig. 2D; Table). To determine if luminol-based bioluminescence could be detected in inflamed eyes, PMU was initiated in 4 animals. On the day of peak inflammation (2 days after intravitreal injection) bioluminescence was generated with an intraperitoneal injection of 200 mg/kg luminol sodium salt. Baseline bioluminescence in flux (photons/second) was compared to bioluminescence at peak inflammation in the treated (right) and control (left) eye (Fig. 2G). The average bioluminescence in treated eyes at peak inflammation (1.46 × 10^5^ photons/second [p/s]) was significantly increased over baseline (1.47 × 10^4^ p/s (*P* = 0.01). There was no difference in bioluminescence in control eyes at baseline and on day 2.

**Table:**
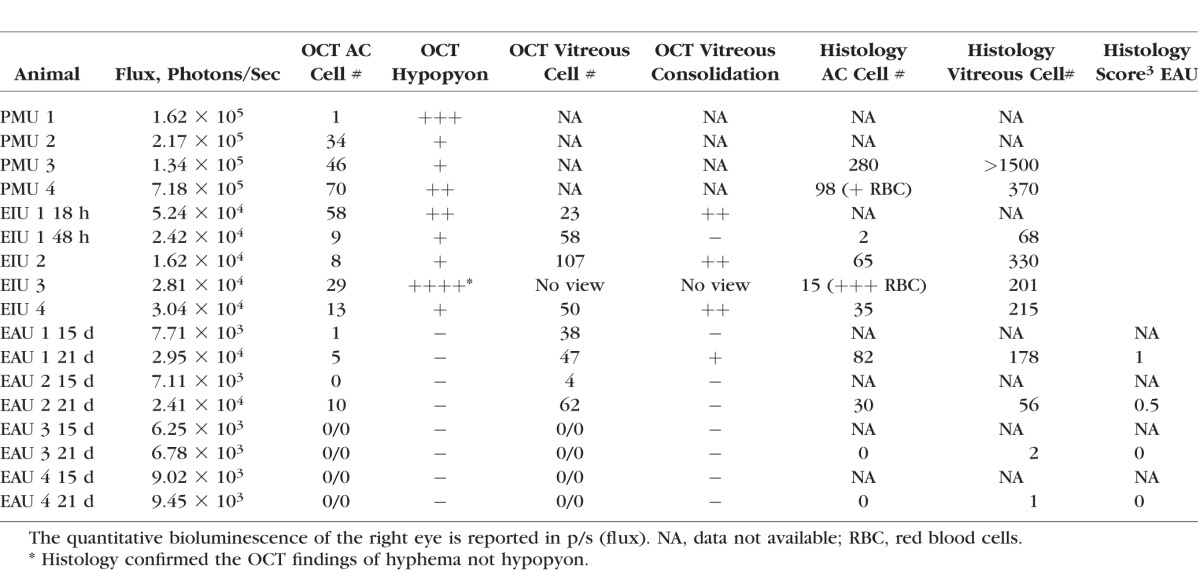
Results of Quantitative Bioluminescence, OCT, and Histology Scores

Endotoxin-induced uveitis is a hyperacute form of anterior and intermediate uveitis that can be generated with systemic or intraocular injection of LPS, and has a significant infiltrate of CD45+, Ly6G+, Cd11b+ neutrophils at peak inflammation.^12,19^ This is a common model of uveitis that may be a good approximation of the mechanisms underlying HLA-B27–associated uveitis in humans.^20^ Endotoxin-induced uveitis was generated by intraocular injection of LPS and bioluminescence was determined at baseline before injection and 18 hours later (peak inflammation). At 18 hours after LPS injection, the average bioluminescence of 4 treated eyes (3.18 × 10^4^ p/s) was significantly increased over baseline (1.09 × 10^4^ p/s, *P* = 0.04). There was no difference in bioluminescence in control eyes (Fig. 3J). Serial imaging of the inflamed and control eye of one animal (Fig. 3K), revealed a 4-fold increase in bioluminescence at 18 hours that returned to near baseline levels at 48 hours. Optical coherence tomography imaging revealed decreased hypopyon (Figs. 3B, 3C) but persistent vitritis (Figs. 3E, 3F) in the inflamed eye at 48 hours.

Both PMU and EIU are models of anterior and intermediate uveitis. To determine if luminol-based bioluminescence also could be applied to detection of inflammation in a model of subacute posterior uveitis, we initiated EAU in 4 albino animals. Right eyes were imaged by OCT and IVIS on days 0, 15, and 21. On day 15, there was OCT evidence of inflammation including vitritis (Fig. 4E) and rare AC cell (Fig. 4B), in 2 of the 4 animals. However, this was not reflected by an increase in the bioluminescence signal from either animal. On day 21, there was an increase in corneal thickness, AC cells, vitreous cells, and disruption of outer retinal layers including retinal folds in the same two animals that had demonstrated signs of inflammation on day 15 (Figs. 4C, 4F). Bioluminescence also increased at day 21 in these animals (2.85 × 10^4^ and 2.41 × 10^4^ p/s) over baseline levels (2.4 × 10^3^ and 7.08 × 10^3^ p/s). In contrast, two animals showed no evidence of inflammation on day 21 by OCT or histology, and there was no increase in bioluminescence when compared to baseline (Fig. 4K; Table).

**Figure 5:**
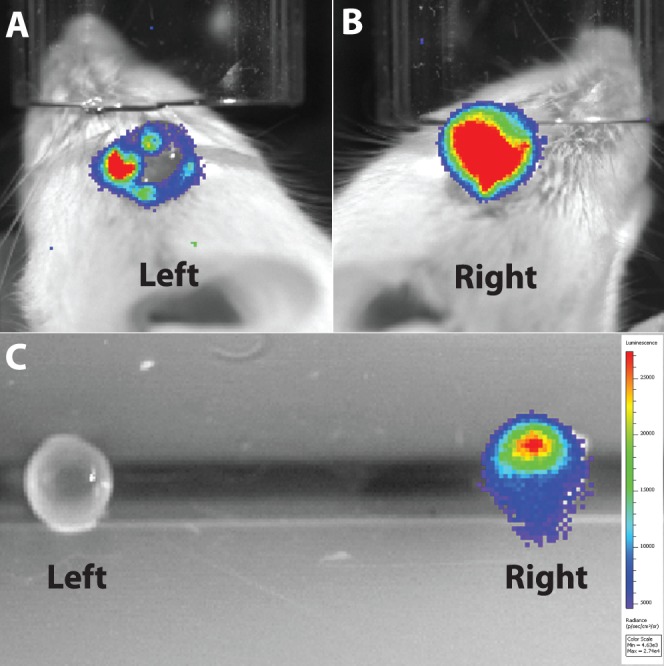
Ex vivo imaging eliminates nonspecific conjunctival bioluminescence in a control eye. Imaging of a PMU animal on day 2. (**A**) Control left eye shows unexpected bioluminescence. (**B**) Inflamed right eye shows the expected high levels of bioluminescence. (**C**) Repeat imaging after enucleation and removal of extraocular tissue.

In general, the bioluminescent signal was localized to the experimental eye. However, one animal showed an exception. Figure 5 shows day 2 images of an animal that had PMU induced in the right eye. This animal was imaged during early IVIS protocol development and troubleshooting stages and was not included among the experimental pilot animals in Figure 2. While the inflamed eye had a strong signal consistent with other PMU animals (Fig. 5B), there also was a signal generated from the control eye (Fig. 5A). The pattern of the bioluminescence, a ring on the outer border of the eye, suggested that the signal might be coming from the conjunctiva on the ocular surface rather than from inside the eye. The animal was reinjected with Luminol and killed for ex vivo imaging. The eyes were enucleated with careful dissection of periocular tissue and conjunctiva, and IVIS imaging was repeated (Fig. 5C). The ex vivo images demonstrate that only the treated eye has an intraocular bioluminescent signal, and supports the conclusion that the bioluminescent signal in the control eye was dependent on the conjunctiva or other extraocular structure. The removed tissue was not evaluated by histology, so we cannot be sure what generated this result, but our suspicion is that the animal developed conjunctivitis and this led to the unexpected bioluminescence. Further studies to develop this method will be required to determine the extent that conjunctivitis or other nonspecific ocular surface injuries will contribute to confounding results with this method.

## Discussion

This study demonstrated that intraocular inflammation is detectable using luminol-based bioluminescence imaging in three models of uveitis. Designed as a pilot feasibility study, these experiments were not powered to detect small differences in bioluminescence with inflammation. Nonetheless in PMU and EIU (both acute models with robust anterior inflammation) significant changes in bioluminescence were observed at peak inflammation over baseline. Experimental autoimmune uveitis (a chronic posterior uveitis) generated a less robust signal. This could be due to the low levels of clinical inflammation that were generated in the albino C57BL/6 strain with the 1-20 IRBP peptide. More robust EAU inflammation has been reported when using the 160 to 181 IRBP peptide,^21^ and could be tested for improved signal in the future. Alternatively, the low levels of bioluminescence in EAU could be due to fewer myeloperoxidase-containing cells in EAU compared to EIU and PMU. Overall, this pilot study demonstrated the feasibility of using IVIS bioluminescence as a quantifiable assay that could be used for monitoring longitudinal inflammation in some animal models of uveitis.

The next step required in the development of this tool will be the correlation of the levels of inflammation by well-established clinical endpoints with the inflammation measured by bioluminescence. In this study we used a combination of in vivo OCT imaging and postmortem histology to verify the presence of inflammation in association with a positive bioluminescent signal. Qualitatively, eyes with PMU generated the most robust signs of inflammation with significant AC cellular reaction, pupillary membrane formation, and corneal edema. Correspondingly, the bioluminescence was 10-fold more intense then that detected in EIU and EAU. At this time it is not clear if this is a purely a manifestation of the overall number of cells in the inflammatory infiltrate, the anatomic location of the infiltrate, or a reflection of the types of cells entering the eye. The luminol-dependent bioluminescence signal has been demonstrated to be dependent on myeloperoxidase in vitro, and in vivo in a mouse model of dermatitis.^8^ Myeloperoxidase is the most abundant protein component of the azurophilic granules of neutrophils and is present in the lysosome of monocytes.^22^ Neutrophils and inflammatory monocytes have been identified in inflamed eyes in the mouse models of EIU^19^ and EAU^23^ by flow cytometry and in the rat model of PMU^14^ by immunohistochemistry. One recent comparative study reported nearly 70% of CD11b+ cells in EIU eyes were neutrophils while only 1% were found in EAU eyes.^23^ This likely contributes to the difference in bioluminescence signal generated by these two models. The primary oxidizing agent that interacts with luminol to generate the bioluminescence signal is believed to be hypochlorous acid generated by the activity of myeloperoxidase, but other oxidizing agents may be generated in these models of uveitis that contribute to the bioluminescence signal. Repeating these studies in a myeloperoxidase mutant would help clarify if additional oxidizing agents are contributing to the signal.

There are differences in the visual representation of inflammation seen in the color distribution of the photon heat maps. Photons appear to be concentrated “within” the eye (Fig. 2B) when the highest photon intensity (red) is well centered on the eye. Other images have distributions that are less well centered raising the possibility that they come from “outside” of the eye (Fig. 4I). It is not clear what these differences signify. Two possible explanations include differences in transpupillary versus trans-scleral photon transmission, or artifact from long exposure window (eye or head movement). Bioluminescence generated within the eye theoretically should radiate out in all directions, but due to the optical properties of the eye many photons likely will be captured by the lens and focused to generate heat maps like those seen in Figure 2. However inflammation from the anterior retina or ciliary body could generate photons that pass directly through the scleral wall forming a signal adjacent to the limbus. Another possibility is that over the 10-minute imaging window, slight eye or head movements could make the collected photons appear to have been generated “outside” of the eye when overlaid on the black and white snapshot obtained at the outset of the imaging session. With strong evidence of intraocular inflammation by OCT and histology, we included all potential signal from the eye by setting the region of interest for quantitative analysis to include the entire orbit in baseline and follow up images.

One of the inherent limitations to bioluminescence imaging is the impact of tissue depth and pigmentation on photon transmission. Luminance generated more than 1 cm below the surface can be very difficult to detect.^16^ The eye avoids this problem due to its superficial location. However, uveal pigment may present a significant barrier to transmission of light from within the eye. Of note, we performed these experiments in albino animals, and transmission in pigmented strains will need to be established. An additional limitation is that, depth of the luminescence signal cannot be determined precisely so localization is limited. Finally, as opposed to fluorescence imaging modalities where longer imaging windows can be used to detect weaker signals, there is a limit on the length of the bioluminescent imaging window that is dependent on the metabolism and clearance of luminol. In this study we followed standard IVIS protocols to determine that the optimal imaging window occurred between 10 and 20 minutes after IP injection of luminol. After the peak imaging window, there is a drop off in photon detection such that longer imaging provides limited increase in signal over background. It is possible that repeated injection, or other methods providing longer exposure to luminol could be used to increase the sensitivity to detect lower levels on inflammation (such as in EAU). Further optimization of luminol dosing, detection specifications, and image analysis parameters could be pursued to improve IVIS protocols for uveitis studies. In vivo bioluminescence is unlikely to replace modalities, such as OCT, but has great potential to become a more widely used complimentary assay.

New imaging techniques are advancing the field of experimental uveitis by providing reproducible, quantitative, and longitudinal methods for measuring and monitoring inflammation in animal models of uveitis.^5,19,24–29^ Optical coherence tomography-based systems have the benefit of providing high-resolution structural images that are amiable to automated quantitative analysis^14,19,24^ or that can be combined with in vivo imaging of fluorescently labeled ocular structures in transgenic mice.^30^ However, media opacity generated by ocular inflammation (corneal edema, cataract, hypopyon, vitritis) can degrade OCT image quality and limit quantitative analysis. In contrast, bioluminescence has the potential to provide quantitative data despite the presence of ocular media opacity.^7^ Bioluminescence-based systems also provide the opportunity for high throughput studies as commercial CCD systems like the IVIS have the capacity to image up to five animals at one time. Furthermore, genetic options exist to generate bioluminescence using transgenic expression of the luciferase enzyme and exogenous administration of the bioluminescence substrate D-luciferin.^6^ Transgenic mice carrying luciferase reporters coupled to immune cell-specific promoters (T-cell, B-cell, Neutrophil, etc.) could provide an in vivo quantitative assay for the relative contribution of subsets of inflammatory cells to acute inflammation, during spontaneous resolution, and in response to therapy.^7^ Currently, this level of specificity requires postmortem immunohistochemistry or flow cytometry analysis.

In summary, this pilot study demonstrated the feasibility of using bioluminescence as a quantifiable assay for use in longitudinal monitoring of ocular inflammation in animal models of uveitis.

## References

[1] Caspi RR. Understanding autoimmune uveitis through animal models. The Friedenwald Lecture. *Invest Ophthalmol Vis Sci*. 2011; 52: 1872–1879. 21450922 10.1167/iovs.10-6909 PMC3101683

[2] Dick AD, Cheng YF, McKinnon A, Liversidge J, Forrester JV. Nasal administration of retinal antigens suppresses the inflammatory response in experimental allergic uveoretinitis. A preliminary report of intranasal induction of tolerance with retinal antigens. *Br J Ophthalmol*. 1993; 77: 171–175. 8457510 10.1136/bjo.77.3.171 PMC504465

[3] Agarwal RK, Silver PB, Caspi RR. Rodent models of experimental autoimmune uveitis. *Methods Mol Biol*. 2012; 900: 443–469. 22933083 10.1007/978-1-60761-720-4_22 PMC3810964

[4] Thurau SR, Chan CC, Nussenblatt RB, Caspi RR. Oral tolerance in a murine model of relapsing experimental autoimmune uveoretinitis (EAU): induction of protective tolerance in primed animals. *Clin Exp Immunol*. 1997; 109: 370–376. 9276535 10.1046/j.1365-2249.1997.4571356.x PMC1904752

[5] Xu H, Koch P, Chen M, Lau A, Reid DM, Forrester JV. A clinical grading system for retinal inflammation in the chronic model of experimental autoimmune uveoretinitis using digital fundus images. *Exp Eye Res*. 2008; 87: 319–326. 18634784 10.1016/j.exer.2008.06.012

[6] Luker KE, Luker GD. Bioluminescence imaging of reporter mice for studies of infection and inflammation. *Antiviral Res*. 2010; 86: 93–100. 20417377 10.1016/j.antiviral.2010.02.002 PMC2863000

[7] Sadikot RT, Blackwell TS. Bioluminescence imaging. *Proc Am Thorac*. 2005; 2: 537–540. 10.1513/pats.200507-067DS PMC2713342 16352761

[8] Gross S, Gammon ST, Moss BL, Bioluminescence imaging of myeloperoxidase activity in vivo. *Nat Med*. 2009; 15: 455–461. 19305414 10.1038/nm.1886 PMC2831476

[9] Stevens P, Winston DJ, Van Dyke K. In vitro evaluation of opsonic and cellular granulocyte function by luminol-dependent chemiluminescence: utility in patients with severe neutropenia and cellular deficiency states. *Infect Immun*. 1978; 22: 41–51. 215546 10.1128/iai.22.1.41-51.1978 PMC422113

[10] Rose S, Waters EA, Haney CR, Meade CT, Perlman H. High-resolution magnetic resonance imaging of ankle joints in murine arthritis discriminates inflammation and bone destruction in a quantifiable manner. *Arthritis Rheum*. 2013; 65: 2279–2289. 23740612 10.1002/art.38030 PMC4002267

[11] Yokota K, Saito T, Kobayakawa K, The feasibility of in vivo imaging of infiltrating blood cells for predicting the functional prognosis after spinal cord injury. *Sci Rep*. 2016; 6: 25673. 27156468 10.1038/srep25673 PMC4860707

[12] Rosenbaum JT, McDevitt HO, Guss RB, Egbert PR. Endotoxin-induced uveitis in rats as a model for human disease. *Nature*. 1980; 286: 611–613. 7402339 10.1038/286611a0

[13] Willermain F, Rosenbaum JT, Bodaghi B, Interplay between innate and adaptive immunity in the development of non-infectious uveitis. *Prog Retin Eye Res*. 2012; 31: 182–194. 22120610 10.1016/j.preteyeres.2011.11.004 PMC3288447

[14] Choi WJ, Pepple KL, Zhi Z, Wang RK. Optical coherence tomography based microangiography for quantitative monitoring of structural and vascular changes in a rat model of acute uveitis in vivo: a preliminary study. *J Biomed Opt*. 2015; 20: 16015. 10.1117/1.JBO.20.1.016015 PMC4296737 25594627

[15] Mruthyunjaya P, Khalatbari D, Yang P, Efficacy of low-release-rate fluocinolone acetonide intravitreal implants to treat experimental uveitis. *Arch Ophthalmol*. 2006; 124: 1012–1018. 16832025 10.1001/archopht.124.7.1012

[16] Chen H, Thorne SH. Practical methods for molecular in vivo optical imaging. *Curr Protoc Cytom*. 2012; 59: 12.24.1–12.24.11. 25419262 10.1002/0471142956.cy1224s59 PMC4240620

[17] Pepple KL, Rotkis L, Van Grol J, Primed mycobacterial uveitis (PMU): histologic and cytokine characterization of a model of uveitis in rats. *Invest Ophthalmol Vis Sci*. 2015; 56: 8438–8448. 26747775 10.1167/iovs.15-17523 PMC4699411

[18] Cheng CK, Berger AS, Pearson PA, Ashton P, Jaffe GJ. Intravitreal sustained-release dexamethasone device in the treatment of experimental uveitis. *Invest Ophthalmol Vis Sci*. 1995; 36: 442–453. 7843913

[19] Chu CJ, Gardner PJ, Copland DA, Multimodal analysis of ocular inflammation using the endotoxin-induced uveitis mouse model. *Dis Model Mech*. 2016; 9: 473–481. 26794131 10.1242/dmm.022475 PMC4852501

[20] Rosenbaum JT. Why HLA-B27? My thirty-year quest: the Friedenwald lecture. *Invest Ophthalmol Vis Sci*. 2011; 52: 7712–7715. 21960642 10.1167/iovs.11-8247 PMC3183985

[21] Mattapallil MJ, Silver PB, Cortes LM, Characterization of a new epitope of IRBP that induces moderate to severe uveoretinitis in mice with H-2b haplotype. *Invest Ophthalmol Vis Sci*. 2015; 56: 5439–5449. 26284549 10.1167/iovs.15-17280 PMC4544201

[22] Schultz J, Kaminker K. Myeloperoxidase of the leucocyte of normal human blood. I. Content and localization. *Arch Biochem Biophys*. 1962; 96: 465–467. 13909511 10.1016/0003-9861(62)90321-1

[23] Liyanage SE, Gardner PJ, Ribeiro J, Flow cytometric analysis of inflammatory and resident myeloid populations in mouse ocular inflammatory models. *Exp Eye Res*. 2016; 151: 160–170. 27544307 10.1016/j.exer.2016.08.007 PMC5053376

[24] Pepple KL, Choi WJ, Wilson L, Van Gelder RN, Wang RK. Quantitative assessment of anterior segment inflammation in a rat model of uveitis using spectral-domain optical coherence tomography. *Invest Ophthalmol Vis Sci*. 2016; 57: 3567–3575. 27388049 10.1167/iovs.16-19276 PMC4942250

[25] Harimoto K, Ito M, Karasawa Y, Sakurai Y, Takeuchi M. Evaluation of mouse experimental autoimmune uveoretinitis by spectral domain optical coherence tomography. *Br J Ophthalmol*. 2014; 98: 808–812. 24574437 10.1136/bjophthalmol-2013-304421

[26] Chen J, Qian H, Horai R, Chan CC, Caspi RR. Use of optical coherence tomography and electroretinography to evaluate retinal pathology in a mouse model of autoimmune uveitis. *PLoS One*. 2013; 8: e63904. 23691112 10.1371/journal.pone.0063904 PMC3653843

[27] Yamamoto T, Goto H, Yamakawa N, Kinetics of polymorphonuclear leukocytes in an experimental hypopyon model. *Exp Eye Res*. 2010; 91: 685–690. 20723542 10.1016/j.exer.2010.08.012

[28] Downie LE, Stainer MJ, Chinnery HR. Monitoring of strain-dependent responsiveness to TLR activation in the mouse anterior segment using SD-OCT. *Invest Ophthalmol Vis Sci*. 2014; 55: 8189–8199. 25414189 10.1167/iovs.14-15595

[29] Chu CJ, Herrmann P, Carvalho LS, Assessment and in vivo scoring of murine experimental autoimmune uveoretinitis using optical coherence tomography. *PLoS One*. 2013; 8: e63002. 23690973 10.1371/journal.pone.0063002 PMC3653962

[30] McNabb RP, Blanco T, Bomze HM, Method for single illumination source combined optical coherence tomography and fluorescence imaging of fluorescently labeled ocular structures in transgenic mice. *Exp Eye Res*. 2016; 151: 68–74. 27519152 10.1016/j.exer.2016.08.003 PMC5072542

